# Translational Informatics for Natural Products as Antidepressant Agents

**DOI:** 10.3389/fcell.2021.738838

**Published:** 2022-01-20

**Authors:** Rajeev K. Singla, Shikha Joon, Li Shen, Bairong Shen

**Affiliations:** ^1^ Institutes for Systems Genetics, Frontiers Science Center for Disease-related Molecular Network, West China Hospital, Sichuan University, Chengdu, China; ^2^ iGlobal Research and Publishing Foundation, New Delhi, India

**Keywords:** depression, natural products, translational informatics, clinical applications, natural antidepressants, neurological disorder

## Abstract

Depression, a neurological disorder, is a universally common and debilitating illness where social and economic issues could also become one of its etiologic factors. From a global perspective, it is the fourth leading cause of long-term disability in human beings. For centuries, natural products have proven their true potential to combat various diseases and disorders, including depression and its associated ailments. Translational informatics applies informatics models at molecular, imaging, individual, and population levels to promote the translation of basic research to clinical applications. The present review summarizes natural-antidepressant-based translational informatics studies and addresses challenges and opportunities for future research in the field.

## Introduction

Depression is a neurological disorder commonly characterized by emotional and physical health, cognitive abilities, behavioral and sleep patterns affecting populations from all age groups globally (Wang et al., 2007; James et al., 2018). Amongst the major contributing factors are the patient’s family and medical history, traumas in early childhood, the structure of the brain, and drug abuse (Wang et al., 2007; James et al., 2018). At present, the medication for depression management largely relies on the chemical-based drugs that are categorized into selective serotonin reuptake inhibitors (SSRIs), serotonin and norepinephrine reuptake inhibitors (SNRIs), tricyclic antidepressants (TCAs), tetracyclic antidepressants, dopamine reuptake blockers, monoamine oxidase inhibitors (MAOIs), 5-HT receptor (5-HT1A, 5-HT2, and 5-HT3) and noradrenergic antagonists (Alvano and Zieher, 2020). The commercially available anti-depressants include citalopram, fluoxetine (SSRIs), desvenlafaxine, duloxetine (SNRIs), amitriptyline (TCAs), maprotiline (tetracyclic anti-depressants), isocarboxazid (MAOI), bupropion (dopamine reuptake blockers), vilazodone, nefazodone, and vortioxetine (5-HT1A, 5-HT2, and 5-HT3 receptor antagonists), and mirtazapine (noradrenergic antagonist) (Anonymous, 2000; Milev et al., 2016), which are accompanied by potential side-effects. Precisely, these vary from common side-effects (nausea, drowsiness, fatigue, constipation, dry mouth, weight gain, trouble sleeping, nervousness, tremors, and sexual problems) to serious health complications (low blood pressure, irregular heartbeat, seizures) (Ferguson, 2001). It is, therefore, imperative to venture into the natural products-based therapeutic domains.

Plant and natural products-based traditional medicine provides the foundation for numerous commercial drugs and has played a significant role in addressing global primary health requirements, especially, in developing countries (Duke et al., 1993; UNESCO World Decade for Cultural Development, and Organization W.H, 1996; Cragg et al., 1997; Newman et al., 2003; OrganizationW.H, 2013; Jyoti and Kumar., 2019; Mukhtar and Singh, 2020; Newman and Cragg, 2020; Atanasov et al., 2021; Dangar and Patel, 2021; Sathya and Arumugam, 2021). These natural products are isolated from various sources that include animals, fungi, plants, and microbes (Gunatilaka, 2006; Cragg and Newman, 2013; Genilloud, 2014; Atanasov et al., 2015; Katz and Baltz, 2016; Newman and Cragg, 2016; Futamura et al., 2017; Ikeda, 2017; Baltz, 2019; Singla et al., 2020; Apoorva et al., 2021; Bansal et al., 2021; Swarnkar et al., 2021). Unlike the chemical commercial drugs, laboratory synthesis of natural products with a heterogeneous composition and unique structures is an arduous task, albeit, enhanced stability and efficacy are reported for them. Moreover, there is often no evidence of microbial resistance, major toxic or side-effects for natural products-based therapeutics, and formulations (Moore et al., 2017). Essentially, these natural products include plant metabolic extracts and secondary metabolites extracted from different medicinal plants. Together, these are reported to demonstrate neuroprotective effects and reduce the risk of neurodegenerative diseases that strengthen their candidature as potential neuroprotective agents (Farahani et al., 2015; Fajemiroye et al., 2016; Martins, 2018; Bhandari et al., 2019). In light of the above facts, ongoing research efforts for depression management are focused on developing natural products-based anti-depressants.

**FIGURE 1:**
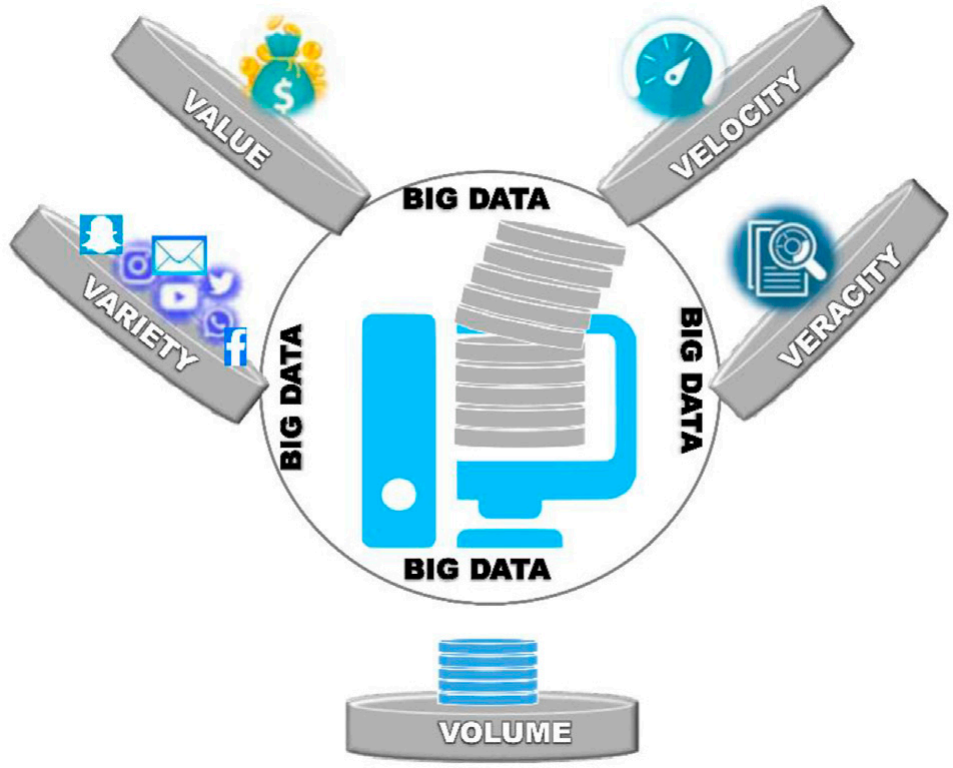
5 Vs of big data characterization for depression.

In the recent past, tremendous progress in the interdisciplinary fields of medicinal science and computer-aided high throughput screening of therapeutic targets has accelerated the ominous process of drug discovery (Singh et al., 2009; Igoli et al., 2014; Singla, 2014; Khan et al., 2020). For this, various therapeutic target databases, models, and tools have been constructed that are essential for the management of vast clinical data (Chen et al., 2020; Liu et al., 2020; Yan et al., 2020; Chen et al., 2021; He et al., 2021). Together, these pave the way to the modern era of drug discovery and “big data,” with a foundation of five pillars,“ namelyValue,” “Variety,” “Velocity,” “Veracity,” and “Volume” (Dhingra and Kumar, 2007). The preclinical and clinical data collected from patients suffering from depression along with the data procured from healthy individuals make up the big depression data. This big data can be utilized for future approaches to data-driven medicine for depression. Figure 1 illustrates the 5 V s of big data characterization for depression. Computer-Aided Drug Discovery (CADD) based on the “big data,” surpasses the traditional methods of drug discovery that rely on ligand and structure-based drug designing tools (Sairam et al., 2002; Hazra et al., 2012; Joon et al., 2021). Howbeit, this demands a standardized multi-tiered data integration with robust algorithms for mining, structuring, and analysis of the accumulated clinical data (Shen et al., 2020; Miao et al., 2021). Also, this is crucial for assessing the precision of drug-target interactions generated by molecular simulation studies. Further, these contemporary drug discovery databases are expected to have ample data storage capacity with proper sources to ascertain the actual data density for the discovery of novel drugs and their targets. Moreover, the appropriate utilization of these modernized computational approaches is crucial for novel drug discovery.

Even though much research has been done on natural product-based therapeutics that strengthen their credibility in the treatment of myriad ailments, the translation of data obtained from basic research to clinical application is still a challenge to the scientific community (Singla and Dubey, 2019; Singla et al., 2021a; Singla et al., 2021b; Singla et al., 2021c; Madaan et al., 2021). With a focus on the antidepressant application of these natural products, here, we propose a paradigm of the informatics-driven research model and summarize the application of informatics models at different levels for augmenting translational research. Since the studies on the application of data models, Bioinformatics, imaging informatics, medical informatics, and health informatics in natural products-based drug discovery is scarce, this review will illuminate developmental insights for future novel translational informatics-based research directions in the field.

## 2 Signaling Pathways of Depression

**FIGURE 2:**
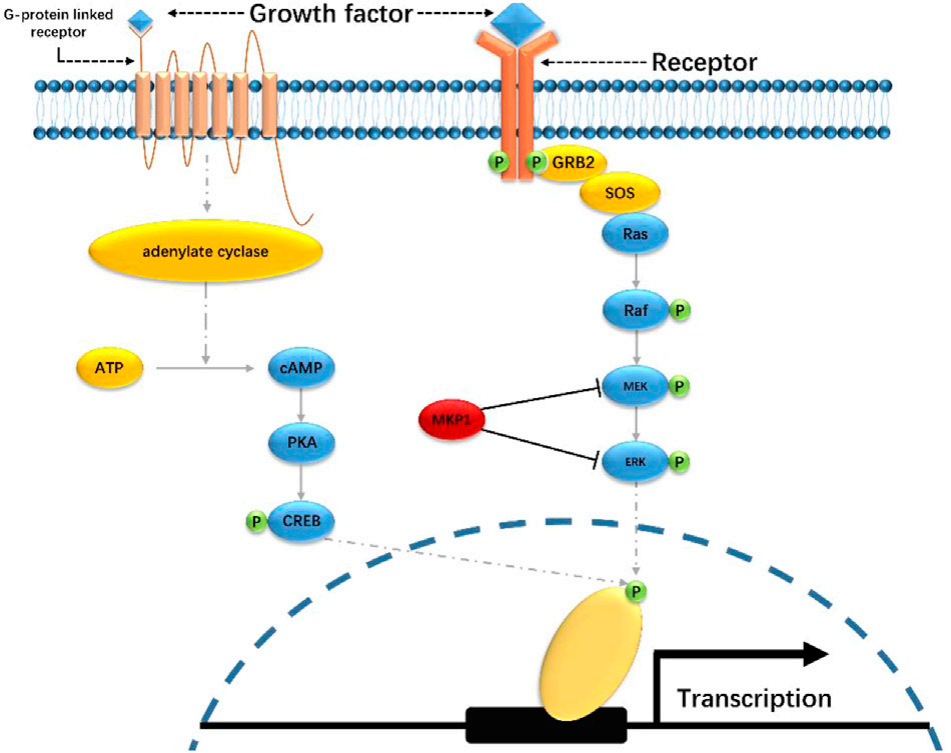
MAPK and cAMP signaling pathways.

Depression pathogenesis involves multiple complex molecular mechanisms (Figure 2). To date, two pathways, including mitogen-activated protein kinase signaling (MAPK) and cyclic adenosine phosphate signaling (cAMP), have been widely reported to be associated with depression development, which has attracted close attention in antidepressant research (Duman and Voleti, 2012).

The Ras-MAPK signaling pathway includes two key kinases, namely extracellular signal-regulated kinase (ERK) and MAP/ERK kinase (MEK). ERK is typically activated by the phosphorylation of various growth factors. The activated ERK will then enter the nucleus and regulate a bunch of transcription factors, promoting specific genes’ transcription and expression. ERK signaling is regulated by protein kinase A (PKA) and protein kinase C (PKC), the activators of which can activate the ERK 1/2 in the hippocampus (Roberson et al., 1999). Accumulating pieces of evidence have demonstrated that the decreased activity of ERK signaling is one of the contributors to depression. Data from studies on depressive suicide victims showed reduced activity of PKA, PKC, and adenylate cyclase (AC) (Dwivedi et al., 2004). Duric et al. sequenced the hippocampus tissues from major depressive disorder (MDD) patients and noticed a significantly elevated expression of MKP1, which is a suppressor for ERK and MEK (Duric et al., 2010). Moreover, Dwivedi et al. revealed the decreased catalytic and phosphorylation activity of ERK 1/2 via direct examination of the expression level of ERK 1/2 in the brain tissues from the suicide victims suffering from depression (Dwivedi et al., 2001). They also noticed the downregulation of Ras, which is an upstream regulator of ERK, and the interaction between Ras and MEK1 is limited as well (Dwivedi et al., 2009). Besides, MAPK suppressors like protein tyrosine phosphatase receptor type R (PTPRR) are also engaged in the ERK-involved depression mechanisms. Overexpression of PTPRR in mice led to their susceptibility to depression (Li et al., 2016). Taken together, ERK signaling plays a crucial role in enhancing neuron plasticity and promoting the release of neural growth factors. The general mechanisms of ERK signaling in depression development are due to its inactivation or suppression. Studies from a system level, however, should be addressed to further explore the landscape of ERK-engaged depression pathogenesis.

The cAMP signaling, or cAMP/AC/PKA signaling, is heavily involved in emotion regulation. A general route of this pathway starts from the activation of AC, which will result in the catalysis of ATP to cAMP, followed by the activation of PKA. The activated PKA will then phosphorylate the cAMP response element-binding protein (CREB) and finally regulate the gene transcription. Studies have noticed that the promotion of cAMP signaling may have antidepressant effects. For instance, Data from Jiang et al. showed that the immobility time of rats was significantly reduced by a PKA agonist named 8-BR-camp (Jang et al., 2018). Nico et al. used a cAMP analog that inhibits PKA but not cAMP and revealed that besides the activation of PKA, the elevation of cAMP can also promote the activity of this signaling pathway and enhance the antidepressant effects (Liebenberg et al., 2011). In addition, Wang et al. also showed similar results. By injection of Raleigh Alvin (rolipram), which is an inhibitor for phosphoric acid lipase 2–4 (PDE4) that can prevent the hydrolysis of cAMP, the researchers found the increased level of cAMP and phosphorylated CREB, and reduce of immobile time of the mice (Wang et al., 2019). Notably, the cAMP and MAPK signaling partially share the same proteins in depression development. There may be interactions between these two pathways, the relationship of which should be paid more attention to, especially for those antidepressant relevant research.

## 3 Natural Products Against Depression

The traditional system of medicine is the bedrock for several commercial drugs for depression and is based on natural products from various sources (Baird-Lambert et al., 1982; England, 1998; Hu et al., 2002; Hedner et al., 2006; Kochanowska et al., 2008; Zhao et al., 2016; Kochanowska-Karamyan et al., 2020; Singla, 2021). There is substantial evidence on the antidepressant activity of metabolic extracts and metabolites isolated from various medicinal plants (Farahani et al., 2015). The metabolic extracts are derived from distinct plant parts, such as leaves, flowers, roots, fruits (powdered or unripe), stem bark, bulb (powdered), whole plant, seed, petal, stigma, rhizome, hypocotyls, and etc. Reportedly, the plant secondary metabolites with anti-depression activity belong to different classes of phytocompounds that mainly comprise alkaloids, flavonoids, furocoumarins, glycosides, polyphenols, saponins, triterpenoids, and xanthones (Farahani et al., 2015). Together, these execute the anti-depression activity or neuroprotective effects through different mechanisms that target neurological signaling pathways or molecules responsible for depressive disorders (Farahani et al., 2015). The antidepressant properties of some important natural products are discussed below.

### 3.1 Metabolic Extracts

**FIGURE 3:**
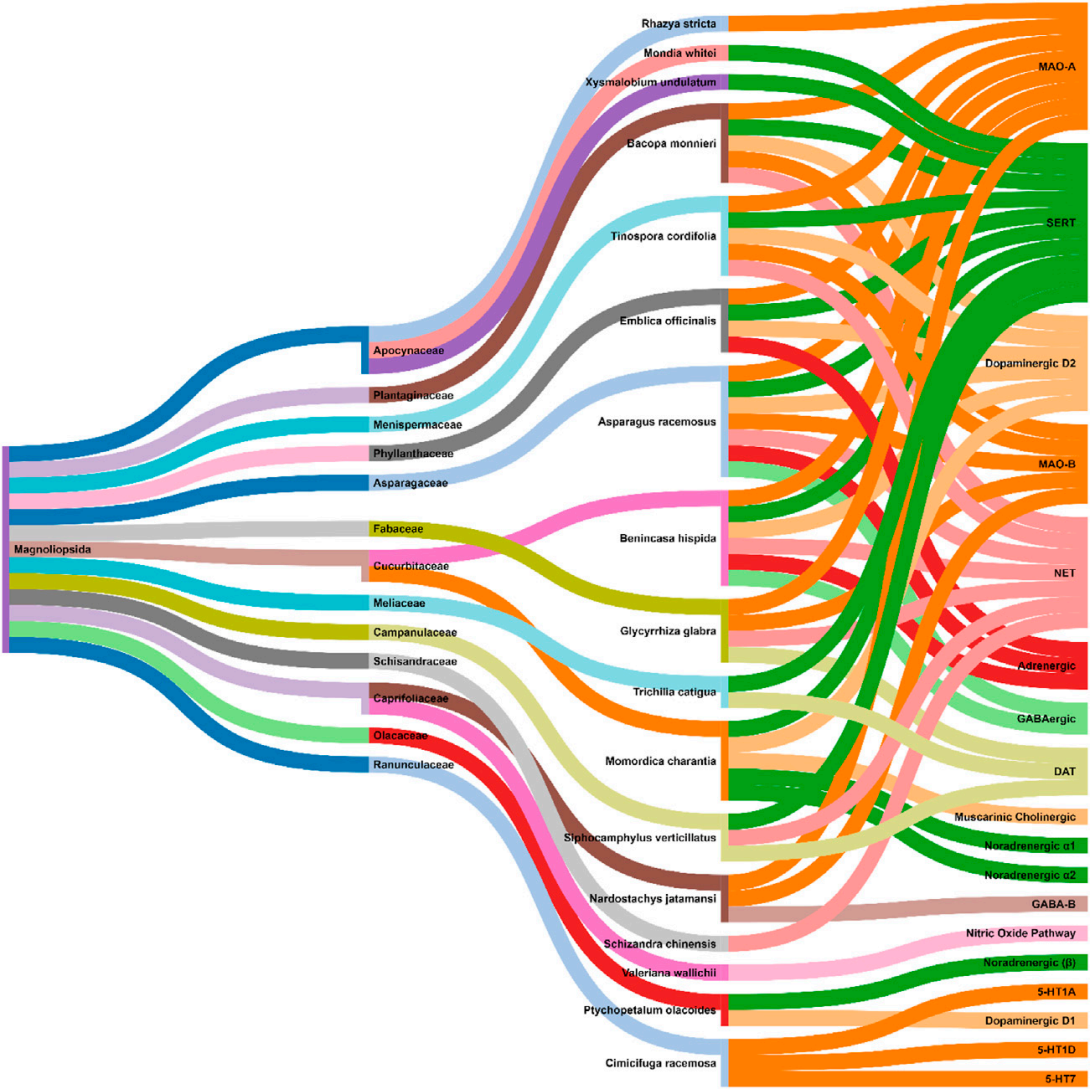
Interaction analysis map to express association and relationship between class and family of natural sources with the physiological pathways related to depression.

The methanolic extracts of *Asparagus racemosus* Willd. (roots) demonstrated *in vivo* antidepressant effects via MAO (Monoamine oxidases: MAO-A and MAO-B) inhibitory activity and dopaminergic (D2), serotonergic, GABAergic (Gamma-aminobutyric acid**)**, adrenergic (α1), and noradrenergic receptor system interactions (Dhingra and Kumar, 2007; Singh et al., 2009). Similarly, the whole plant extracts of *Bacopa monnieri* (L.) Wettst. exerted *in vivo* antidepressant effects mainly through MAO (Monoamine oxidases: MAO-A and MAO-B) inhibitory activity and dopaminergic (D2), noradrenergic, and serotonergic receptor system interactions (Sairam et al., 2002; Maity et al., 2011; Hazra et al., 2012; Singh et al., 2014; Girish et al., 2016). Further, the fruit (methanolic) and seed (aqueous) extracts of *Benincasa hispida* (Thunb.) Cogn. exhibited MAO-A enzyme inhibition activity and dopaminergic (D2), serotonergic, GABAergic, adrenergic, and noradrenergic receptor system interactions (Dhingra and Joshi, 2012; Bharti and Singh, 2013). *Phyllanthus emblica* L. aqueous fruit extract showed MAO-A inhibitory activity and dopaminergic (D2), serotonergic, adrenergic receptor system interactions. This antidepressant activity could be due to the ascorbic acid, tannins, flavonoids, and polyphenols present in its fruit (Pemminati et al., 2010; Dhingra et al., 2011). Another plant extract that acts through MAO (MAO-A and MAO-B) activity inhibition is *Glycyrrhiza glabra* L. The aqueous, hydroalcoholic and ethanolic root extracts of *G. glabra* L. elevates norepinephrine (NE) and dopamine (DA) levels in the brain (Dhingra and Sharma, 2006; Chowdhury et al., 2011; Biswas et al., 2012). The petroleum ether stem extracts of *Tinospora cordifolia* (Willd.) Hook. f. and Thomson demonstrated antidepressant activity by MAO (MAO-A and MAO-B) enzyme inhibitory activity and dopaminergic (D2), serotonergic, adrenergic, and noradrenergic receptor interactions (Dhingra and Goyal, 2008a). The ethanolic and aqueous leaf extracts of *Rhazya stricta* Decne. demonstrated antidepressant effects through MAO-A inhibition (Ali B. et al., 1998; Ali B. H. et al., 1998). *Momordica charantia* L. relies on dopaminergic (D2), serotonergic (5-HT2), muscarinic, cholinergic, and noradrenergic (α1 and α2) receptor systems for its antidepressant activity (Ishola et al., 2013). Piato et al. and Siqueira et al. showed that *Ptychopetalum olacoides* Benth. (ethanolic root extract) possess antidepressant activity along with the noradrenergic (β) and dopamine (D1) receptor system interactions (Siqueira et al., 2004; Piato et al., 2009). Pedersen et al. demonstrated that *Mondia whitei* (Hook.f.) Skeels and *Xysmalobium undulatum* (L.) W.T.Aiton possess antidepressant activities, which could be attributed to their affinity for the Serotonin transporter (SERT) (Pedersen et al., 2008). Reportedly, the ethanolic root and rhizome extracts of *Nardostachys jatamansi* (D.Don) DC. showed anti-MAO (MAO-A and MAO-B) activity and GABAB receptor interaction (Dhingra and Goyal, 2008b; Karanth et al., 2012; Deepa et al., 2013). The root and rhizome extracts of (aqueous, dichloromethane, hydroethanolic, oil, and methanolic) *Valeriana jatamansi* Jones ex Roxb. were reported to have antidepressant effects via inhibition of nitric oxide (Subhan et al., 2010; Sah et al., 2011a; Sah et al., 2011b). Viana et al. demonstrated the antidepressant activity of *Schisandra chinensis* (Turcz.) Baill. (seeds) to be through its interaction with noradrenergic receptors (Viana et al., 2005). Rodrigues et al. showed that the hydroethanolic stem and leaf extracts of *Siphocamphylus verticillatus* possess antidepressant activity via synaptosomal inhibitory activity. These include [3H] dopamine [3H] noradrenaline, and [3H] serotonin uptake (Rodrigues et al., 2002). The uptake of serotonin and dopamine was also inhibited by the hydroethanolic bark extract of *Trichilia catigua* A. Juss. (Campos et al., 2005). Burdette et al. showed antidepressant activity of various extracts (aqueous, ethanol, and isopropanol) of *Actaea racemosa* L. It was reported to behave as a serotonin (5-HT1A, 5-HT1D, and 5-HT7) receptor agonist (Burdette et al., 2003). Figure 3 shows an interaction analysis map of metabolite extracts from various plant sources with the physiological biomarkers of depression. Except for *Rhazya stricta* Decne., *Mondia whitei* (Hook.f.) Skeels*, Valeriana jatamansi* Jones ex Roxb.*, Schisandra chinensis* (Turcz.) Baill.*,* and *Xysmalobium undulatum* (L.) W.T.Aiton, the rest all the metabolite extracts were documented to be multimodal in action for their antidepressant activity. To the surprise, all the natural sources belong to a single class (Magnoliopsida). Further, as per the covered data, the family Apocynaceae, Curcurbitaceae, and Caprifoliaceae were of special importance to explore natural antidepressants. In our previous literature study, we have observed that families like Solonaceae and Fabaceae, are important in yielding agents against Parkinson’s disease (Singla et al., 2021a).

### 3.2 Metabolites

**FIGURE 4:**
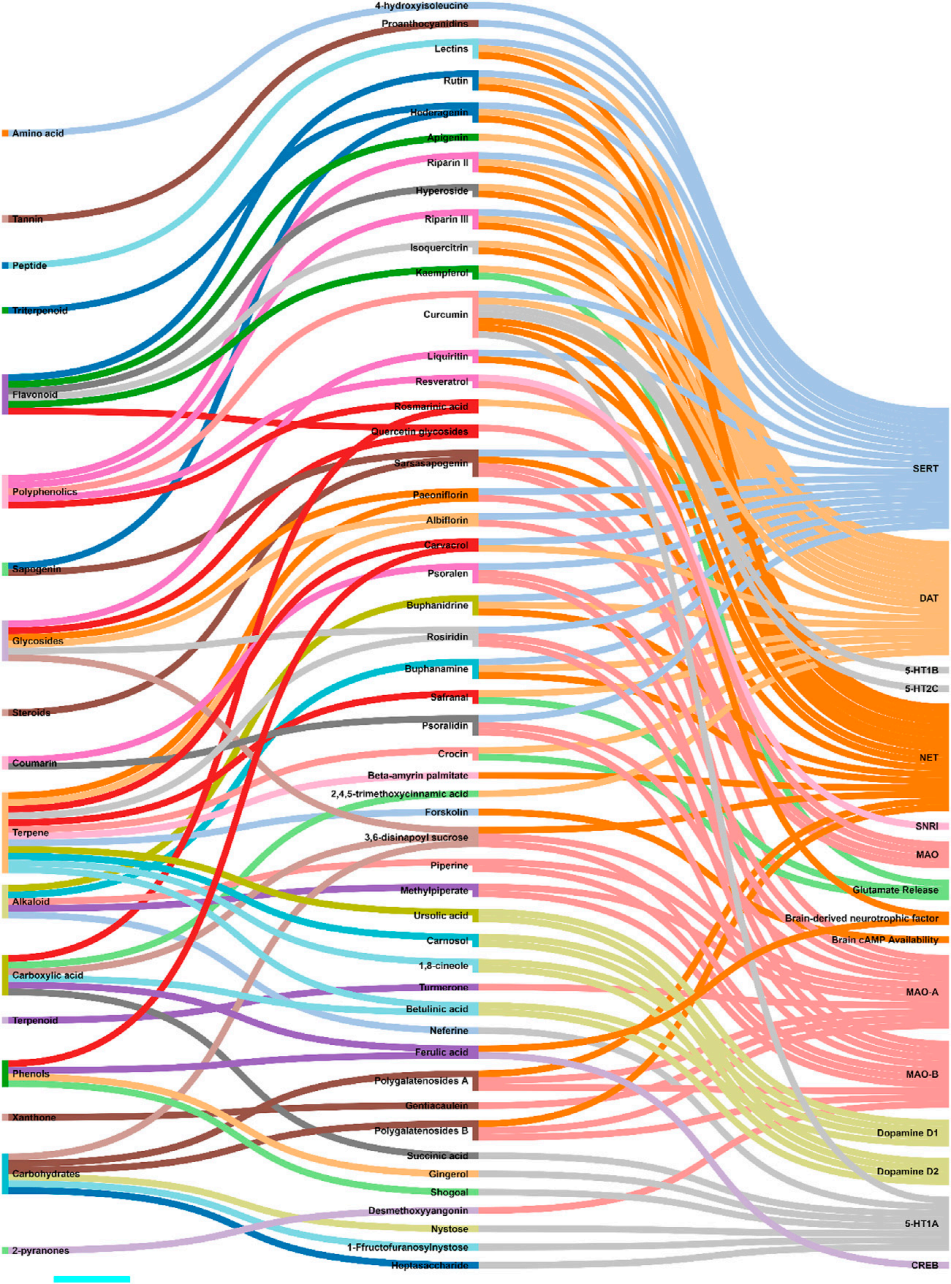
Interaction analysis map to express association and relationship between phytochemical classifications of antidepressant metabolites with the physiological pathways related to depression.

**FIGURE 5:**
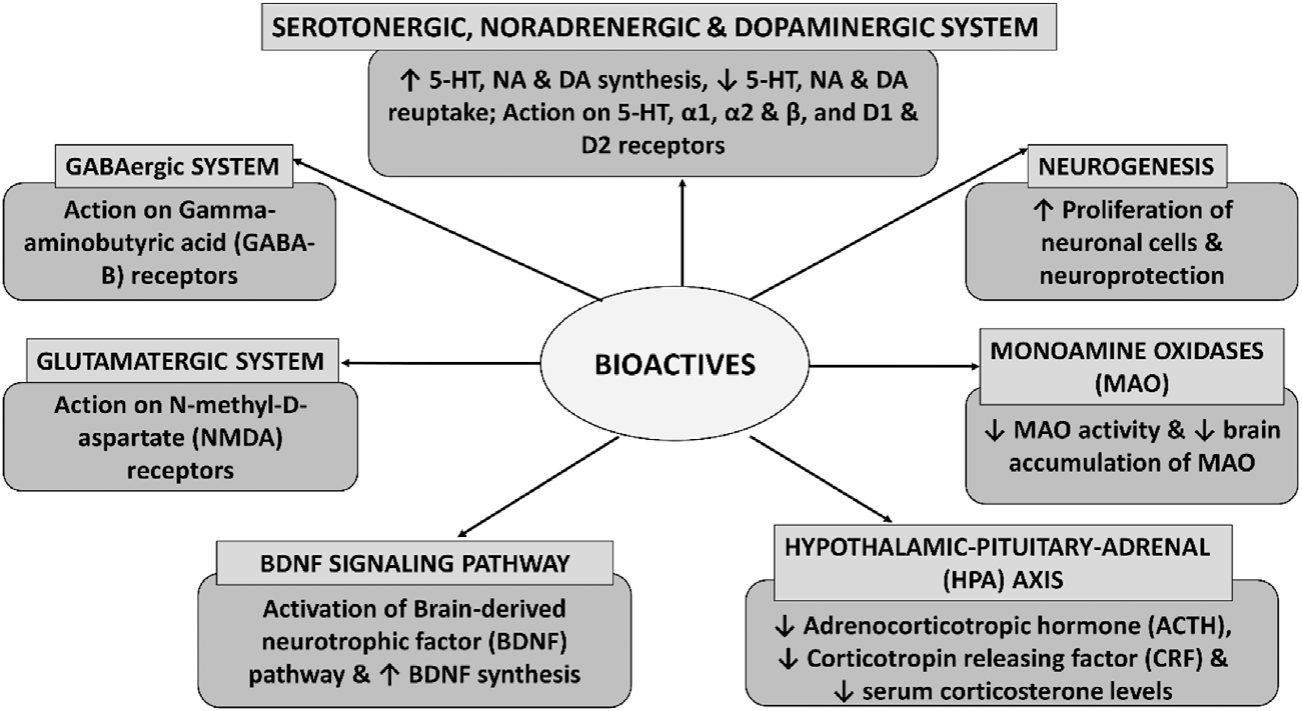
Illustration of the molecular mechanisms mediated by the bioactives for depression management.

Zhou et al. and Jin et al. demonstrated that antidepressant effects of *Akebia trifoliata* (Thunb.) Koidz. (ethanolic powdered fruit extract) are due to hederagenin (Supplementary Figure S1A), which has high norepinephrine transporter (NET), dopamine transporter (DAT), and serotonin (SERT) transporter affinity, and inhibitory activity (humans and rats) (Zhou et al., 2010; Jin et al., 2012). Likewise, several studies reported anti-NET, anti-DAT, and anti-SERT activities of alkaloids, namely, buphanamine (Supplementary Figure S1B) and buphanidrine (Supplementary Figure S1C), isolated from the ethanolic bulb extract of *Boophone disticha* (L.f.) Herb. (Nielsen et al., 2004; Pedersen et al., 2008; Gadaga et al., 2010). Quercetin (Supplementary Figure S1D) isolated from the aqueous powdered bulb extract of *Allium cepa* L. demonstrated MAO inhibitory activity. An increased metabolite to neurotransmitter ratio was observed in rat models of depression administered with quercetin (Sakakibara et al., 2014). Likewise, paeoniflorin (Supplementary Figure S1E) and albiflorin (Supplementary Figure S1F) isolated from the ethanolic root extract of *Paeonia lactiflora* Pall. exhibited MAO inhibitory activity. These glycosides also up-regulated the serotonergic systems (Mao Q.-Q. et al., 2008; Mao Q. et al., 2008; Qiu et al., 2013). Ren et al. showed that sarsasapogenin (Supplementary Figure S1G) isolated from *Anemarrhena asphodeloides* Bunge leaf extracts possessed MAO (MAO-A and MAO-B) inhibitory activity. It was also shown to interact with norepinephrine and serotonin (5-HT) receptor systems (Ren et al., 2006). MAO inhibitory activity was observed with piperine (Supplementary Figure S1H) and methylpiperate (Supplementary Figure S1I) isolated from the ethanolic fruit extract of *Piper longum* L. (Lee et al., 2005; Lee et al., 2008). Other examples include polygalatenoside A (Supplementary Figure S1J) and polygalatenoside B, YZ-50, and 3,6-disinapoyl sucrose (Supplementary Figure S1K) isolated from the root extracts of *Polygala tenuifolia* Willd. that caused NE-mediated MAO activity inhibition (Cheng et al., 2006; Hu et al., 2010; Hu et al., 2011). Isoliquiritin (Supplementary Figure S1L) and liquiritin (Supplementary Figure S1M) isolated from the aqueous root extracts of *Glycyrrhiza uralensis* Fisch. ex DC. demonstrated serotonin (enhanced 5-HT) and NE-mediated antidepressant activity in the mice model of depression (Wang W. et al., 2008; Zhao et al., 2008; Fan et al., 2012). 4-hydroxyisoleucine (Supplementary Figure S1N) isolated from the seed extract of *Trigonella foenum-graecum* L. exhibited serotonergic system-mediated antidepressant activity (Gaur et al., 2012). Various other metabolites, such as 1 F-fructofuranosylnystose (Supplementary Figure S1O), heptasaccharide (Supplementary Figure S2P), inulin-type hexasaccharide, nystose (Supplementary Figure S2Q), succinic acid (Supplementary Figure S2R) (aqueous root extracts of *Gynochthodes officinalis* (F.C.How) Razafim. and B. Bremer) (Cui et al., 1995; Zhang et al., 2002), neferine (Supplementary Figure S2S) (seed extracts of *Nelumbo nucifera Gaertn*) (Dhanarasu and Al-Hazimi, 2013), gingerol (Supplementary Figure S2T) and shogoal (hydromethanolic rhizome extract of *Zingiber officinale* Roscoe) (Pratap et al., 2012; Sibi and Meera, 2013) were demonstrated to be agonists for serotonin receptor (5-HT1A). Turmerone (Supplementary Figure S2U) (aqueous rhizome extract of *Curcuma longa* L.) (Yu et al., 2002; Liao et al., 2013) and gentiacaulein (Supplementary Figure S2V) (diethyl ether aerial extract of *Gentiana acaulis* L.) (Tomic et al., 2005) impeded MAO-A activity while desmethoxyyangonin (Supplementary Figure S2W) and pyrones (*Piper methysticum* G. Forst.) caused dopaminergic-dependent MAO-B inhibition *in vivo* (Baum et al., 1998; Uebelhack et al., 2007). Furocoumarins, namely, psoralen (Supplementary Figure S2X) and psoralidin (Supplementary Figure S2Y) isolated from the seed extract of *Cullen corylifolium* (L.) Medik. possessed serotonergic-dependent MAO inhibitory activity (Xu et al., 2008; Yi et al., 2008). Similar antidepressant activities were observed with rosiridin (Supplementary Figure S2Z) isolated from the aqueous, dichloromethane, and methanolic root extracts of *Rhodiola rosea* L. (Supplementary Figure S2) (van Diermen et al., 2009; Mannucci et al., 2012). Riparins (Riparin II (Supplementary Figure S2AA) and Riparin III (Supplementary Figure S2AB) (Sousa et al., 2004; Teixeira et al., 2013), lectins (Barauna et al., 2006), and rutin (Supplementary Figure S2AC) (Machado et al., 2007; Machado et al., 2008) isolated from the unripe fruit (ethanolic extract), seed, and stem and leaf (hexane and ethanolic extracts) of *Aniba riparia* (Nees) Mez, *Canavalia brasiliensis* Mart. ex Benth., and *Schinus molle L*. respectively were shown to interact with dopaminergic, noradrenergic, and serotonergic receptor systems. β-amyrin palmitate (Supplementary Figure S2AD) isolated from the methanolic leaf extracts of *Lobelia inflata* L. showed antidepressant activity via noradrenergic receptor system activation (Subarnas et al., 1992; Subarnas et al., 1993). Flavonoids, precisely, hyperoside (Supplementary Figure S2AE) and isoquercitrin (Supplementary Figure S2AF) isolated from the ethanolic leaf extracts of *Apocynum venetum* L. caused elevated hippocampus levels of NE and DA. Dopaminergic receptor system (D1 and D2) interactions were observed (Butterweck et al., 2001; Zheng et al., 2013). 1,8-cineole (Supplementary Figure S2AG), betulinic acid (Supplementary Figure S2AH), carnosol (Supplementary Figure S2AI), and ursolic acid (Supplementary Figure S2AJ) isolated from *Salvia rosmarinus* Spenn. (stem and leaf extracts) demonstrated antidepressant effect via dopamine receptor activation (Machado et al., 2012; Machado et al., 2013; Mukhtar et al., 2013; Singla et al., 2017). 2,4,5-trimethoxycinnamic acid (Supplementary Figure S2AK), apigenin (Supplementary Figure S3AL), and rosmarinic acid (Supplementary Figure S3AM) isolated from the leaves of *Perilla frutescens* (L.) Britton demonstrated dopaminergic system-dependent antidepressant activity (Nakazawa et al., 2003; Ito et al., 2008; Yi et al., 2013). Carvacrol (Supplementary Figure S3AN) (aromatic plant extract) caused a dopaminergic system-mediated antidepressant effect leading to elevated levels of serotonin (5-HT) and dopamine (Melo et al., 2011; Zotti et al., 2013). On the contrary, curcumin (Supplementary Figure S3AO) exerts its antidepressant activity via the serotonergic receptor system (5-HT1A/1B and 5-HT2C) causing an elevation in the serotonin (5-HT) levels (Wang R. et al., 2008; Kulkarni et al., 2008). The otherwise altered 5-HT1A mRNA (hippocampus) was also reversed in curcumin-treated mice models of depression (Xu et al., 2007). Resveratrol (Supplementary Figure S3AP) showed MAO inhibitory activities and increased dopamine, noradrenaline, and serotonin (5-HT) levels in rat models of depression (Xu et al., 2010; Yu et al., 2013). Crocin (Supplementary Figure S3AQ), kaempferol (Supplementary Figure S3AR), and safranal (Supplementary Figure S3AS) isolated from the petal and stigma extracts (aqueous and ethanolic extracts) of *Crocus sativus* L. demonstrated a potential antidepressant activity by inducing the release of brain dopamine and glutamine (Hosseinzadeh et al., 2004; Hosseinzadeh et al., 2007; Ettehadi et al., 2013). Forskolin (Supplementary Figure S3AT) isolated from *Coleus hadiensis* (Forssk.) A.J.Paton showed antidepressant activity by enhancing the availability of cAMP in the brain (Wachtel and Loschmann, 1986; Maeda et al., 1997). Ferulic acid (Supplementary Figure S3AU) increases CREB phosphorylation and mRNA levels of a brain-derived neurotropic factor in mice models of depression (Yabe et al., 2010). The scientific name of the medicinal plants was mentioned as per the universally accepted nomenclature, specified and recommended by the Ethnopharmacology team. So, the names specified in the manuscript will seems to be different from that of cited articles. To cross-check the nomenclature, refer Medicinal Plant Names Service (MPNS) https://mpns.science.kew.org/mpns-portal/and http://www.plantsoftheworldonline.org/. Data for Figures 3, 4 was collected manually by literature search using PubMed and Google Scholar. For Figure 3, the taxonomical class of the biological sources has been retrieved from the NCBI taxonomy browser. For Figure 4, the phytochemical class was mentioned as per the classification mentioned in PubChem, NCBI. Then all these data were transformed as per the Sankey Graph principles to convert into an interactive illustration. Figure 4 shows an interaction analysis map of various potential antidepressant phytochemicals or metabolites with the physiological biomarkers of depression. It has been observed that the majority of natural antidepressants fall under the category of carbohydrates, glycosides, phenols, polyphenolics, flavonoids, carboxylic acids, and terpenes. Figure 5 illustrates the molecular mechanisms mediated by the bioactives for depression management.

### 3.3 Natural Products From Various Other Sources for Depression Management

Karamyan et al. demonstrated *in vivo* antidepressant activity for veranamine isolated from the marine sponge (*Verongula rigida*). Owing to its selective affinity towards sigma-1 and 5HT2B receptors, it could serve as a novel antidepressant drug candidate (Kochanowska-Karamyan et al., 2020). Similarly, barettin, 8,9 dihydrobarettin, gelliusines A and B, and sigma-conotoxin have been reported to possess selective affinity towards the serotonin receptors (England, 1998; Hedner et al., 2006). Further, Lambert et al. reported the MAO inhibitory activity for methylaplysinopsin isolated from *Aplysinopsis reticulate* (a sponge) (Baird-Lambert et al., 1982). The antagonist binding displacement activity at 5HT_2A_ and 5HT_2C_ receptors have been reported for the compounds isolated from *Smenospongia aurea* (a sponge) (Hu et al., 2002). There are some other marine natural products reported to possess promising antidepressant activity *in vivo* (Kochanowska et al., 2008). For example, potential *in vivo* antidepressant activity was reported for the total sterols and β-sitosterol isolated from *Sargassum horneri* (a brown seaweed). There occurred a significant increase in NE, 5-HT, and 5-HIAA (5-hydroxyindoleacetic acid) neurotransmitters (Zhao et al., 2016).

## 4 Databases Comprising Natural Products—Sharing of Data and References

With the breakthrough technologies, such as omics and informatics, biomedical research has made significant strides (Canuel et al., 2014; McGuire et al., 2020). Since the collected biomedical information is huge, data management with highly organized databases is enormously important (Harel et al., 2011). Besides, such well-structured databases must be amenable to sharing and integration of the standardized and annotated stored data (Misra et al., 2019). Natural products comprise a spectrum of potential therapeutic compounds for myriad diseases. Owing to the spectacular diversity in natural products, the development of natural-products databases is essential (Mehbub et al., 2014; Atanasov et al., 2015; Cheesman et al., 2017). These databases, in turn, will broaden our horizon on the mechanistic insights of natural products or compounds on a particular disease target and reveal crucial clinical details for ‘precision medicine’. One such example is COCONUT, an acronym for MongoDB COlleCtion of Open Natural prodUcTs (https://coconut.naturalproducts.net) that comprise freely accessible natural products databases, albeit partially (Sorokina et al., 2021).

### Traditional Medicine Databases

Based on the geographical location, the traditional medicinal system has various distinct branches with shared attributes. To name a few, these conventional medicinal systems include traditional Indian medicine, traditional Chinese medicine, and traditional Islamic medicine (Pan et al., 2014; Yuan et al., 2016). From a holistic perspective, the development of robust databases and knowledge bases is imperative for a systematic sharing and annotation of these traditional medicinal systems that encompass enormous information on natural products. This indeed is indispensable for an integrated evaluation and screening of natural products (Ikram et al., 2015). Over the years, endeavours have been undertaken to build compendious natural products databases that accommodate essential data on the natural products, their potential targets, and genetic interactions. HERB (http://herb.ac.cn) and SymMap (https://www.symmap.org/) are exemplary traditional Chinese medicine databases. HERB contains elementary information on herbs with their putative targets and genetic interaction mechanisms while SymMap is useful in mapping the disease symptoms disseminating appropriate prescriptions (Wu et al., 2019; Fang et al., 2021). Others in the category are the curated Indian Medical Plants, Phytochemistry And Therapeutics (IMPPAT) (https://cb.imsc.res.in/imppat), and Universal Natural Product Resource (UNaProd) (http://jafarilab.com/unaprod) databases that provide useful information on the nomenclature and medicinal applications of Indian and Iranian herbs, respectively (Mohanraj et al., 2018; Naghizadeh et al., 2020). Noteworthy, data on natural products contained within these traditional medicine databases may provide us with valuable conventional clinical and therapeutic anti-depression prescriptions. Howbeit, a real-time integrated data analysis is all-important for gaining newer clinical insights to the global scientific community dedicated to depression research.

### Databases of Different Natural Product Sources

In recent times, there has been a resurgence in interest in natural product-based drug discovery research (Gu et al., 2013; Laganà et al., 2019). However, this demands prompt, apt, and credible screening methods for natural products, followed by their isolation from the heterogeneous extracts and structural characterization to strengthen their therapeutic potential over alternate drug discovery processes. Additionally, these screening tools must be competent for the large-scale production of natural products-based therapeutic compounds (Butler, 2004). It is, therefore, incumbent on the global research community to develop robust and comprehensive natural products databases that comprise crucial information on the species source and quantitative pharmacological activity of all possible natural products, in addition to their structural details and qualitative pharmacological activity. Unfortunately, the existing general and specialized natural products databases disseminate experimental quantitative activity data for the few natural products contained within. The examples include SuperNatural (http://bioinformatics.charite.de/supernatural) (Banerjee et al., 2015), ZINC (http://zinc.docking.org/) (Irwin and Shoichet, 2005), TCM-ID (Traditional Chinese Medicine Information Database) (http://bidd.group/TCMID/) (Wang et al., 2005), TCM@Taiwan/iSMART (SysteMs Biology Associated Research with TCM) (http://ismart.cmu.edu.tw/) (Chang et al., 2011), TCMID (Traditional Chinese Medicines Integrated Database) (http://119.3.41.228:8000/tcmid/) (Huang L. et al., 2018), TCMSP (Traditional Chinese Medicine Systems Pharmacology Database and Analysis Platform) (https://old.tcmsp-e.com/tcmsp.php) (Ru et al., 2014), TM-MC (Northeast Asian traditional medicine) (http://informatics.kiom.re.kr/compound) (Kim et al., 2015), NuBBE_DB_ (Nuclei of Bioassays, Ecophysiology and Biosynthesis of Natural Products Database) (https://nubbe.iq.unesp.br/portal/nubbedb.html) (Pilon et al., 2017), SANCDB (South African Natural Compounds Database) (https://sancdb.rubi.ru.ac.za/) (Hatherley et al., 2015), HIT (Herbal ingredients’ targets databases) (https://bio.tools/hit) (Ye et al., 2010), NPACT (Naturally Occurring Plant-based Anti-cancer Compound-Activity-Target database (http://crdd.osdd.net/raghava/npact/) (Mangal et al., 2013), and BioPhytMol (http://ab-openlab.csir.res.in/biophytmol/) (Sharma et al., 2014). To this end, numerous natural products databases were constructed to complement the existing databases with ample information on species sources and experimental quantitative activity for myriad natural products. These include NPASS (Natural Product Activity and Species Source) (http://bidd2.nus.edu.sg/NPASS/) (Zeng et al., 2018), NANPDB (Northern African Natural Products Database) (http://african-compounds.org/nanpdb/) (Ntie-Kang et al., 2017), and SuperNatural II (Super Natural database) (http://bioinformatics.charite.de/supernatural) (Banerjee et al., 2015). These provide an enhanced knowledge of the structural and physicochemical attributes for a large majority of natural compounds along with their toxicity class prediction, metabolic pathways, pharmacokinetics, biological activity, and related mechanisms, and vendor information (Banerjee et al., 2015; Ntie-Kang et al., 2017; Zeng et al., 2018). Besides, there exist some other natural product databases, such as, NPBS (Natural Products and Biological Sources) (http://www.organchem.csdb.cn/scdb/NPBS) that furnish vital information on the relationship between natural products and their sources (relational data) (Xu et al., 2020). Essentially, the biological source is linked to the natural products derived from it and vice versa. This database with a broader range of natural source species can be exploited to avoid the replication of isolation and characterization of established natural products (Xu et al., 2020). Other examples include CMNPD (Comprehensive Marine Natural Products Database) (https://www.cmnpd.org/) (Lyu et al., 2021) and PAMDB (*Pseudomonas Aeruginosa* Metabolome Database) (http://pseudomonas.umaryland.edu/) (Huang W. et al., 2018) that encompass data on marine natural products and the metabolic pathway diagrams and metabolomics on *Pseudomonas aeruginosa*, respectively. There are some others that include, PSC-db (http://pscdb.appsbio.utalca.cl) (Valdés-Jiménez et al., 2021), TeroKit (http://terokit.qmclab.com) (Zeng et al., 2020), MedPServer (http://bif.uohyd.ac.in/medserver) (Potshangbam et al., 2018), TriForC (http://bioinformatics.psb.ugent.be/triforc) (Miettinen et al., 2018), 3DMET (http://www.3dmet.dna.affrc.go.jp) (Maeda and Kondo, 2013), BiG-FAM (https://bigfam.bioinformatics.nl) (Kautsar et al., 2021), DEREP-NP (https://github.com/clzani/DE) (Zani and Carroll, 2017), TMDB (http://pcsb.ahau.edu.cn:8080/TCDB/index.jsp) (Yue et al., 2014). In all, these databases with an enormous wealth of information on natural products and their source species (biological sources) might be instrumental in augmenting the efforts directed towards screening of anti-depression therapeutics, drug discovery, and development. Table 1 outlines the databases comprising natural products-sharing of data and references as discussed above.

**TABLE 1:** Databases comprising natural products-sharing of data and references.

Databases	Specification	Website	PMID	References
COCONUT	Comprise freely accessible natural products databases	https://coconut.naturalproducts.net	33423696	Sorokina et al. (2021)
Traditional medicine databases				
HERB	Contains elementary information on herbs with their putative targets and genetic interaction mechanisms	http://herb.ac.cn	33264402	Fang et al. (2021)
SymMap	SymMap is useful in mapping the disease symptoms for appropriate prescriptions	https://www.symmap.org/	30380087	Wu et al. (2019)
IMPPAT	Provide information on the nomenclature and medicinal applications of Indian herbs	https://cb.imsc.res.in/imppat	29531263	Mohanraj et al. (2018)
UNaProd	Provide information on the nomenclature and medicinal applications of Iranian herbs	http://jafarilab.com/unaprod	32454857	Naghizadeh et al. (2020)
Databases of different natural product sources				
Supernatural II	Provides segregated information on natural products, their 2D structures, structural and physicochemical characteristics, toxicity prediction, and associated pathways for natural product synthesis, their degradation and activity	http://bioinformatics.charite.de/supernatural	25300487	Banerjee et al. (2015)
TCM-ID	Provides information on natural products for the Traditional Chinese Medicine	http://bidd.group/TCMID/	16003299	Wang et al. (2005)
TCM@Taiwan/iSMART	Allows for virtual screening and drug designing for the Traditional Chinese Medicine using an integrated cloud computing approach	http://ismart.cmu.edu.tw/	21696236	Chang et al. (2011)
TCMID	Provides information on the Traditional Chinese Medicine and interconnections among the herbal constituents, prescriptions, potential targets, drugs, and diseases	http://119.3.41.228:8000/tcmid/	29106634	Huang et al. (2018a)
TCMSP	Provides information on herbal medicines for drug discovery using the systems pharmacology	https://old.tcmsp-e.com/tcmsp.php	24735618	Ru et al. (2014)
TM-MC	Provides information on medicinal materials and chemical compounds used in traditional medicine system of Northeast Asia	http://informatics.kiom.re.kr/compound	26156871	Kim et al. (2015)
NuBBE_DB_	Provides information on natural compounds from the Brazilian biodiversity, including their structure and biological activities	https://nubbe.iq.unesp.br/portal/nubbedb.html	28775335	Pilon et al. (2017)
SANCDB	Provides information on natural compounds from various sources in South Africa	https://sancdb.rubi.ru.ac.za/	26097510	Hatherley et al. (2015)
HIT	Provides information on herbal bioactives and their targets	https://bio.tools/hit	21097881	Ye et al. (2010)
NPACT	Provides information on anti-cancer phytocompounds, their activity and potential targets	http://crdd.osdd.net/raghava/npact/	23203877	Mangal et al. (2013)
BioPhytMol	Provides information on anti-mycobacterial phytocompounds and metabolic extracts from plants for drug discovery	http://ab-openlab.csir.res.in/biophytmol/	25360160	Sharma et al. (2014)
NPASS	Provides information on natural products, their activity and source species	http://bidd2.nus.edu.sg/NPASS/	29106619	Zeng et al. (2018)
NANPDB	Provides information on natural products from various sources in North Africa	http://african-compounds.org/nanpdb/	28641017	Ntie-Kang et al. (2017)
NPBS	Furnish vital information on the relationship between natural products and their sources (relational data)	http://www.organchem.csdb.cn/scdb/NPBS	33306802	Xu et al. (2020)
CMNPD	Provides information on marine natural products	https://www.cmnpd.org/	32986829	Lyu et al. (2021)
PAMDB	Provides information on the metabolic pathway diagrams and metabolomics on *Pseudomonas aeruginosa*	http://pseudomonas.umaryland.edu/	29106626	Huang et al. (2018b)
PSC-db	Provides information on plant secondary metabolites	http://pscdb.appsbio.utalca.cl	33672700	Valdés-Jiménez et al. (2021)
TeroKit	Provides information on terpenome compounds and their properties, facilitates drug discovery of terpenome via implemented toolkits (target profiling and conformer generation modules)	http://terokit.qmclab.com	32286817	Zeng et al. (2020)
MedPServer	Allows for the identification of therapeutic targets and potential natural products leads	http://bif.uohyd.ac.in/medserver	30381914	Potshangbam et al. (2018)
TriForC	Provides information on the plant triterpene biosynthesis	http://bioinformatics.psb.ugent.be/triforc	29045755	Miettinen et al. (2018)
3DMET	Provides information on the 3D structures of natural metabolites	http://www.3dmet.dna.affrc.go.jp	23293959	Maeda and Kondo (2013)
BiG-FAM	Provides information on the biosynthetic gene clusters from microbial and metagenome-assembled genomes	https://bigfam.bioinformatics.nl	33010170	Kautsar et al. (2021)
DEREP-NP	Provides information on natural products from various sources for rapid de-replication, Mass spectroscopy and Fast Nuclear Magnetic Resonance Spectroscopy are exploited for data acquisition	https://github.com/clzani/DE	28616931	Zani and Carroll (2017)
TMDB	Provides information on tea originated small molecular compounds	http://pcsb.ahau.edu.cn:8080/TCDB/index.jsp	25224438	Yue et al. (2014)

## Translational Informatics for Investigation of Potential Natural Products as Anti-depressants—Data Integration and Modeling

Computational tools based on multi-scale modeling (MSM) are explicitly efficient, robust, and dynamic in integrating data, testing hypotheses, and comprehensively illuminating the pathophysiological mechanisms underlying depression-related neurological disorders (Ramirez-Mahaluf et al., 2015; Shen et al., 2019). Collectively, these expedite diagnosis and therapy together with antidepressant target identification for drug development (Ramirez-Mahaluf et al., 2015). These putative antidepressant molecules can be explored to ascertain their candidature as a proficient therapeutic target or biomarker for depression disorders by employing amalgamated and synchronized network-based strategies (Zhang T.-T. et al., 2018; Wu et al., 2018). Over the past few years, deep learning has revolutionized the traditional target screening, which embarks on a new age of drug discovery (Schneider, 2017; Kraus, 2019; Kiriiri et al., 2020; Schaduangrat et al., 2020; Gupta et al., 2021; Paul et al., 2021; Singh et al., 2021). For instance, TripletRes and AlphaFold are globally acclaimed contemporary two-dimensional (2D) protein structure prediction tools of deep learning, which have spectacularly boosted the efficacy of classical drug discovery strategies (Kryshtafovych et al., 2019; Li et al., 2019; Senior et al., 2019). A consolidated yet thoughtful utilization of these modern computational tools will be decisive in exploring naturally occurring molecules for depression management and prognosis (Truax and Romo, 2020; Woo and Shenvi, 2021).

### Computational Models for the Synthesis of Natural Products

In general, the naturally occurring biomolecules are either isolated from microbial fauna or medicinal plants, which is an expensive and labour extensive long rigmarole (Sairam et al., 2002; Sarker and Nahar, 2012; Bucar et al., 2013; Zhang Q.-W. et al., 2018). These cumbersome isolation procedures are oftentimes undermined by inevitable constraints, such as the seasonal variations of plant growth, variations in microbial growth conditions (in the case of microbial biomolecules), the efficacy of the purification procedures employed, and low yields (Sairam et al., 2002; Sarker and Nahar, 2012; Bucar et al., 2013; Zhang Q.-W. et al., 2018). A combination of highly efficient isolation and purification techniques is, therefore, indispensable to obtain these natural products at reasonable yields. Even though there exist a few fully autonomous computational algorithms and tools, these are tarnished by major pitfalls. For instance, these are capable of accomplishing solo commands at a given time and are usually confined to comparatively simple molecular drug targets (Song et al., 2009; Prachayasittikul et al., 2015; Bharatam, 2021). A highly acclaimed synthesis route design tool for complex natural products is Chematica (Mikulak-Klucznik et al., 2020). With the innovation of Chematica, autonomous-computer-aided synthesis pathways are designed swiftly for myriad commercially important natural products and biologically active compounds of medicinal value. In contrast to the previous synthesis pathways, it requires fewer steps with incredible synthetic efficiency and cost-effectiveness evident from its laboratory performance (Klucznik et al., 2018).

It is worth discussing here, that the classical retro-synthesis technique for even simple organic molecules relied on the recursive or repetitive transformation into still smaller entities. This cumbersome task has been hastened by the advent of computer-based retro-synthesis. Quite dismally, these are in their infancy with disappointingly sluggish performance and quality-compromised outputs. Recently, these limitations have been overcome with the introduction of symbolic artificial intelligence (AI) and Monte Carlo tree search-guided revelation of retro-synthesis routes for diverse organic molecules (Segler et al., 2018). This state-of-the-art computer-aided retro-synthesis tool essentially consists of deep neural networks, viz. expansion policy, and filter networks integrated Monte Carlo tree search to allow a guided search with the prior selection of the propitious steps for synthesis route. Further, these deep learning tools are exceptionally swift in their output for diverse molecules over manually designed conventional heuristic methods that rely on extracted rules for synthesis route search. Also, these are well-trained, with an inbuilt knowledge on nearly all the reported organic reactions for myriad molecules as ascertained by a double-blind analysis (Segler et al., 2018).

This spectacular breakthrough in the field of computer-aided retro-synthesis might assist researchers in devising novel techniques to deduce feasible approaches for the optimal synthesis of molecular targets. Also, this does not require any prior knowledge or expertise regarding the existing strategies. Altogether, these contemporary computational tools, perhaps strategize the multi-step complex syntheses route designs for natural products, which are otherwise quite laborious and inefficacious.

### Computational Models for Natural Products-Based “Precision Medicine”

The network-based approaches play a cardinal role in numerous scientific fields (Chandran et al., 2017; Guo et al., 2020). Amongst the various crucial applications is in the domain of biomedical sciences, where it assists in the evaluation of diverse systemic molecular interactions (Sonawane et al., 2019; Sheik Amamuddy et al., 2020; Wang et al., 2020). To perform investigations, such as assessing the effects of dysfunctional molecules in the system as a whole, hitherto biological networks were relied upon (Furlong, 2013; Somvanshi and Venkatesh, 2013; Altaf-Ul-Amin et al., 2014; Charitou et al., 2016; Hu et al., 2016; Caldera et al., 2017; Faeder et al., 2020; Silverman et al., 2020; Wang et al., 2020). The discovery of biomarkers together with a screening of putative drug molecules for complex diseases, including depression-related disorders can be accomplished using more utilitarian network-based applications. These include, but are not limited to co-expression, gene-gene, and protein-protein interaction network-based strategies (Khanin et al., 2011; Vella et al., 2017; Myers et al., 2019; Sun et al., 2019; Ovens et al., 2021).

With the innovation of genomics or genomic sequencing (DNA/RNA), the process of novel drug discovery is tremendously accelerated (Xia, 2017; Suwinski et al., 2019; McGuire et al., 2020). Further, genome sequencing was pioneering in introducing the concept of “Drug repurposing” for medically-approved drugs, which was a turning point in treating diverse ailments and a diminished economic burden for developing newer drugs for individual disease treatment (Emilien, 2000; Jarada et al., 2020; Nabirotchkin et al., 2020). Howbeit, their applicability in the therapeutic management of numerous disparate diseases was sceptical. This limitation is overcome by the contemporary and technologically advanced genomic techniques that sped the identification of specific disease-causing key genetic factors or anomalies in an individual, in particular (Dryja, 1997; Lander et al., 2001; Hasin et al., 2017; Horton and Lucassen, 2019). This “Precision medicine” furnishes a mechanistic insight into an individual patient’s disease (customized disease module) and unveils principal disease contributing elements. Eventually, these disease mechanisms could be targeted with “precision” for high-end personalized treatment strategies (Dugger et al., 2017; König et al., 2017). In this direction, a drug repurposing, Genome-wide Positioning Systems network (GPSnet) algorithm that targets genomic sequence profile-derived disease modules from a single patient was developed. These genomic sequencing profiles allow protein-protein interaction mapping for human diseases that reveal the key molecular players in disease pathophysiology. This, in turn, is pivotal in strategizing and prioritizing the selection of repurposed drugs for an effective and customized treatment regimen. Further, disease modules based on the predictions from GPSnet (https://www.gpsnet.com.br/) could accurately predict responses and strategize usage for a reasonable number of approved chemotherapeutic drugs for approximately five thousand cancer patients on prior *in silico* investigations (transcriptomic profiling and exome sequencing) (Cheng et al., 2019). As a proof of concept, ouabain (cardiac drug) demonstrated an antitumor potency via anti-HIF1α/LEO1 activity *in vitro.* This *in silico* tool could perform the dual role of specifically identifying a disease module and repurposing the approved drugs with precise indications for medical applications as observed in the case of cancer. These findings strengthen the candidature of GPSnet as a drug repurposing scaffold for constructing an effective therapeutic screen for various drugs, including identification of naturally occurring potential drug molecules, their synthesis, and precise administration in depression therapies (Cheng et al., 2019).

There is accumulating evidence on the utility of network theory in evaluating the therapeutic potential of natural products in health management. For instance, association network-based novel techniques were developed for scrutinizing and discerning microbes that synthesize biomolecules from those that participate in the biological transformation of natural (or pharmaceutical) products within the human host. Specifically, these association networks rely on the concomitant probing of metabolomics and metagenomics data on diversified human microbial fauna. As a step further, the intended molecules were mapped to their respective clade and finally to the phylogenetic tree to identify the microbial species participating in their synthesis or biotransformation (Cao et al., 2019). As a futuristic approach, these might supersede the available time and cost-extensive characterization techniques, which are solely dependent on methods of microbial cultivation. Further, this limits their efficiency of species identification that partakes in the synthesis or the transformation of the vivid small molecular wealth found within the host system. The feasibility study of metagenomics and metabolomics association networks unveiled the corynomycolenic acid-producing microbial genes amid the human cystic fibrosis microbiome isolates. Additionally, these accurately delineated the associations of quinolone signals (Pseudomonas), phevalin, and tyrvalin to their respective clusters of biosynthetic genes (Cao et al., 2019).

In another study, the mechanisms that govern natural products and synthetic chemotherapeutic synergism were investigated. Accordingly, the information on compounds and their targets was retrieved from the public domain that aided in assessing the targetable space for respective natural products. In the context of the network, their evaluation accentuated the notion that these natural products exhibit groupings of targets in the family, which are disparate as well as share commonness with a synthetic chemotherapeutic. Conclusively, these rational pieces of evidence emphasize the chemotherapeutic efficiency of natural products for developing complementary and combinatorial novel chemotherapies with synthetic anticancer drugs (Chamberlin et al., 2019). Likewise, multi-potent natural chemotherapeutics were screened from *Clerodendrum indicum* and *C. serratum* using network pharmacology (Gogoi et al., 2017). By employing an integrative approach, the anticancer effects of a combination of drugs against various cancer targets were determined. Amongst the predicted natural anti-cancer compounds, apigenin 7-glucoside and hispidulin could bind efficiently to reasonable chemotherapeutic targets (seventeen). These findings are crucial milestones in the field of novel anti-cancer drug discovery (Gogoi et al., 2017).

Whilst shreds of evidence on network-based approaches for anti-depression natural product discovery are lacking, the above findings might serve as important milestones in bridging the gap in the identification of natural products and developing these as potent anti-depression therapeutics.

## Future Perspectives on Translational Informatics for Investigation of Natural Products Anti-depressants

With a global technological advancement in the healthcare sector, depression management strategies now include translational informatics, which has firmly integrated clinical data with basic research (Smith et al., 2007; Unützer and Park, 2012; Tenenbaum, 2016; Robinson, 2018; Kraus et al., 2019). Also, these aim to strive at “precision medicine” for depression care (Nierenberg, 2012; Menke, 2018; Serretti, 2018). Noteworthy, these substantially rely on multi-tiered databases comprising enormous yet, segregated data from various resources, such as clinical, environmental, lifestyle, and natural products data (Herland et al., 2014; Shameer et al., 2017; Seyhan and Carini, 2019). These, in turn, are essential for developing a well-trained AI system with ample inbuilt knowledge. It is contemplated that the AI system, so developed, can be exploited for performing dual tasks of exploring potential natural therapeutic candidates commenced by recommendation of precise drugs for depression management at first. Secondly, the real-time medical status of a single patient can be monitored based on the instantaneous physiological information together with an automated AI system alarm upon encountering health aberrations. Furthermore, healthcare counselling and related advice can be imparted to patients by day-to-day evaluation using cloud computing. Unfortunately, the AI system for depression management is still in its infancy struggling with issues of privacy and confidentiality of patient information amongst myriad other challenges (Grist et al., 2018; Graham et al., 2019; Hategan et al., 2019; Huckvale et al., 2019; Romano and Tatonetti, 2019; Tran et al., 2019; Bickman, 2020; Ke et al., 2020; Mennen et al., 2021).

**FIGURE 6:**
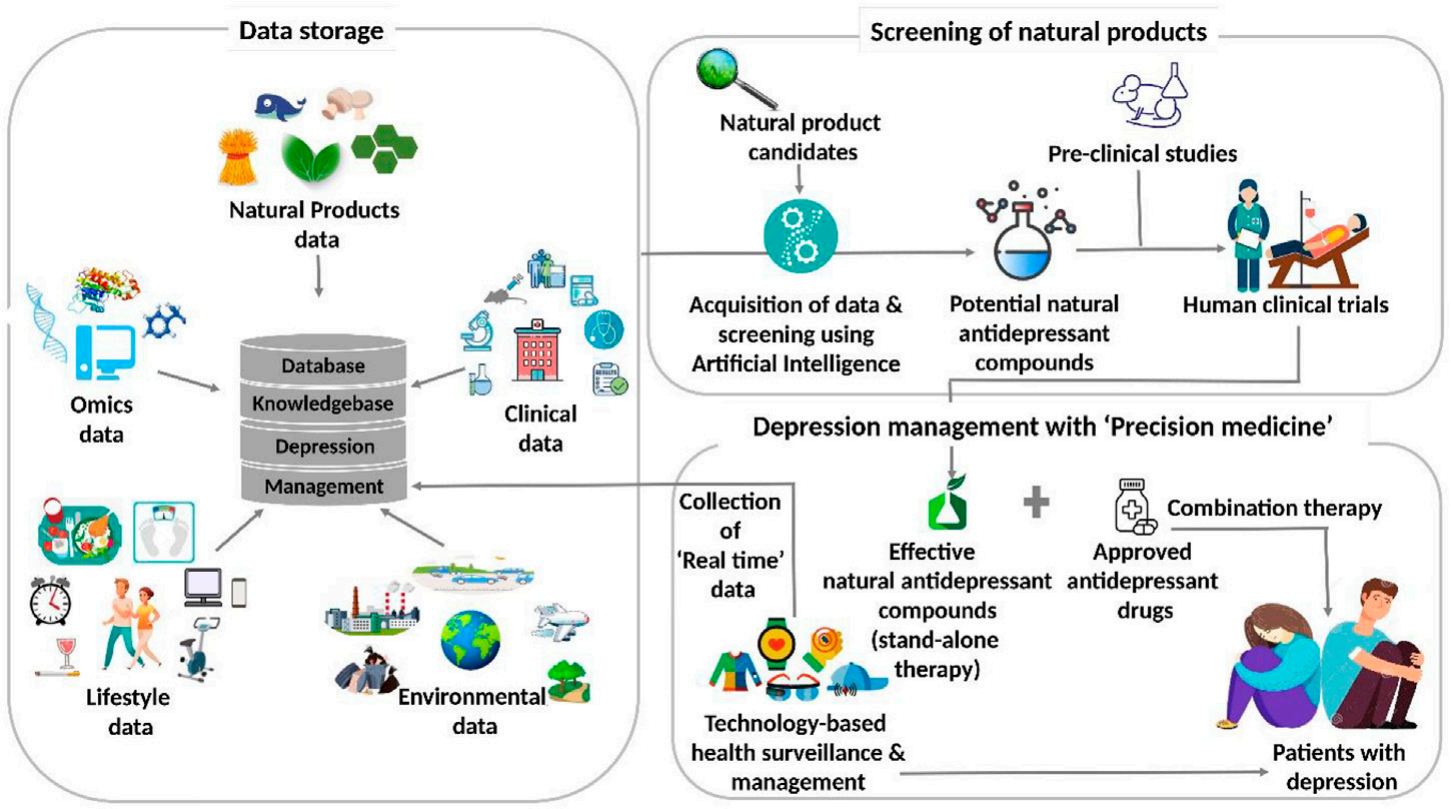
The futuristic translational informatics-based model for depression management using natural antidepressants.

Figure 6 illustrates the futuristic translational informatics-based model for depression management using natural antidepressants. Presently, the databases on natural products store data on the promising candidates for myriad diseases. With the increasing research data on depression-related therapeutics based on natural products and the complexity associated with the disease, it is important to integrate these databases with systematic analysis. These, in turn, are expected to disseminate the references and knowledge for well-trained AI systems. It is, therefore, contemplated that the AI systems can be exploited for performing two important tasks. Firstly, these can be utilized for natural product screening and for endorsing promising candidates for depression-targeted pre-clinical and clinical trials. Next, the effective anti-depression natural products, as revealed by these studies, are included in the treatment regime of the patients diagnosed with depression. Depending upon their effectiveness, these can be administered either as a stand-alone or in combination with commercially approved drugs. Secondly, AI systems can be used for surveillance as well as monitoring the health status of the patients. Accordingly, patients’ physiological data are acquired in real-time through wearables and cloud platform-assisted technology. This caters to the customized needs of the patients by providing them suggestions for their self-care, and their health status is also reported simultaneously. Although this systemic translational informatics-based model for depression management using natural antidepressants is promising and attractive, various issues are yet to be addressed to implement this futuristic approach.

### Databases and Knowledge Bases for Specific Antidepressant Natural Products

Unfortunately, the available databases for natural products contain scarce anti-depression therapeutic candidates (Varteresian and Lavretsky, 2014; Sorokina et al., 2021). As the scientific intrigue and general concern for depression management have gained momentum, it is imperative to develop anti-depression natural therapeutic candidate databases. For futuristic computational therapeutic screening, these depression-specific databases are essential. In light of the disease complexity, these databases must mandatorily employ an integrated systematic analysis. Further, the construction of knowledge databases is suggested as they undertake the compilation of data obtained from distinct levels. These include, but are not limited to, potential therapeutic biomolecules with the databases of natural products, related biomolecules, and their putative targets within the host system, environmental factors, and various other depression-associated attributes. Eventually, a stratified biomedical landscape for depression management is sketched by these knowledge databases. These knowledge databases, in turn, aid in the construction of knowledge graphs, which are expert-populated data integration biomedical resources. Specifically, the biomedical entities (concepts) are illustrated as nodes while inter-entity associations or relationships are depicted as edges (Yu et al., 2017; Nicholson and Greene, 2020). These might be of immense significance in assisting various biomedical applications capable of comprehending novel clinical, genomic, and pharmaceutical details needed for treatment support decisions.

### Systematic Modeling Based on AI Screening of Potential for Antidepressant Natural Products

Since depression is a neurological and debilitating disease with complex traits, which might have genetic roots as observed in the case of other psychiatric disorders such as cystic fibrosis and Huntington’s chorea (Bowcock, 2010; Dunn et al., 2015; Shadrina et al., 2018). Owing to the complexity of contributing factors, the AI system largely reckons on molecular dynamic simulations- and modeling tools-based depression evolutionary analysis for evaluating the implications of natural products for patients with depression disorders (Romano and Tatonetti, 2019). Consequently, unveiling the principal elements and crucial molecular players associated with disease progression and deciphering effective natural products for its management becomes an onerous task. This generates mind-boggling yet critical questions concerning improvisations on the robustness of AI for screening of natural products and precision medicine (Shen et al., 2021). Quite possibly, these can be subdued by employing quality training data, selection, optimization, and validation algorithms, feature extraction as well as standard and validated techniques-based data collection.

### Cross-Level Data Integration-dependent Precision Treatment for Depression

Depression-specific medicinal research encompasses complex clinical and molecular phenotypic data types with yet more complicated interconnections (Krishnan and Nestler, 2008; Clark et al., 2017). By and large, the ongoing research emphasizes certain aspects while neglecting other facets, which might be commensurably important in prompt diagnosis, therapy, and management of depressive disorders. Mapping these entwined clinical and molecular phenotypic linkages is cardinal for modeling in systems biology for any disease type (Schumann et al., 2014). For instance, there have been reports on the therapeutic efficacy of some natural products at the molecular level without any marked effect on the clinical phenotypes of patients (Liu et al., 2015; Yeung et al., 2018). This supports the notion that gathering a paired molecular and clinical data for a defined duration can be instrumental in simulating disease progression, depression, in this case, and generating a reliable model for the same. The Cancer Genome Atlas (TCGA) (https://www.cancer.gov/) and the International Cancer Genome Consortium (ICGC) (https://daco.icgc.org/) are exemplary data integration programs that can be viewed as a landmark for constructing similar depression-disorder programs. In conclusion, Figure 6 represents a promising systemic model for depression management, however, the feasibility of this model relies on several challenges, including the scarce quantitative data on natural antidepressants.
